# Intestinal TGR5 agonism improves hepatic steatosis and insulin sensitivity in Western diet-fed mice

**DOI:** 10.1152/ajpgi.00300.2018

**Published:** 2019-01-03

**Authors:** Patricia D. Finn, David Rodriguez, Jill Kohler, Zhengfeng Jiang, Sindy Wan, Erick Blanco, Andrew J. King, Tao Chen, Noah Bell, Dean Dragoli, Jeffrey W. Jacobs, Rakesh Jain, Michael Leadbetter, Matthew Siegel, Christopher W. Carreras, Samantha Koo-McCoy, Karen Shaw, Cathy Le, Sandra Vanegas, I-Hsin Hsu, Kenji Kozuka, Keiko Okamoto, Jeremy S. Caldwell, Jason G. Lewis

**Affiliations:** Ardelyx, Incorporated, Fremont, California

**Keywords:** hepatic steatosis, intestinal, RDX8940, TGR5, Type 2 diabetes

## Abstract

Takeda G protein-coupled receptor 5 (TGR5) agonists induce systemic release of glucagon-like peptides (GLPs) from intestinal L cells, a potentially therapeutic action against metabolic diseases such as nonalcoholic steatohepatitis (NASH), nonalcoholic fatty liver disease (NAFLD), and Type 2 diabetes. Historically, TGR5 agonist use has been hindered by side effects, including inhibition of gallbladder emptying. Here, we characterize RDX8940, a novel, orally administered TGR5 agonist designed to have minimal systemic effects and investigate its activity in mice fed a Western diet, a model of NAFLD and mild insulin resistance. Agonist activity, binding selectivity, toxicity, solubility, and permeability of RDX8940 were characterized in standard in vitro models. RDX8940 pharmacokinetics and effects on GLP secretion, insulin sensitivity, and liver steatosis were assessed in C57BL/6 mice fed normal or Western diet chow and given single or repeated doses of RDX8940 or vehicle, with or without dipeptidyl peptidase-4 (DPP4) inhibitors. Gallbladder effects were assessed in CD-1 mice fed normal chow and given RDX8940 or a systemic TGR5 agonist or vehicle. Our results showed that RDX8940 is minimally systemic, potent, and selective, and induces incretin (GLP-1, GLP-2, and peptide YY) secretion. RDX8940-induced increases in plasma active GLP-1 (aGLP-1) levels were enhanced by repeated dosing and by coadministration of DPP4 inhibitors. RDX8940 increased hepatic exposure to aGLP-1 without requiring coadministration of a DPP4 inhibitor. In mice fed a Western diet, RDX8940 improved liver steatosis and insulin sensitivity. Unlike systemic TGR5 agonists, RDX8940 did not inhibit gallbladder emptying. These results indicate that RDX8940 may have therapeutic potential in patients with NAFLD/NASH.

**NEW & NOTEWORTHY** Takeda G protein-coupled receptor 5 (TGR5) agonists have potential as a treatment for nonalcoholic steatohepatitis and nonalcoholic fatty liver disease (NAFLD) but have until now been associated with undesirable side effects associated with systemic TGR5 agonism, including blockade of gallbladder emptying. We demonstrate that RDX8940, a potent, selective, minimally systemic oral TGR5 agonist, improves liver steatosis and insulin sensitivity in a mouse model of NAFLD and does not inhibit gallbladder emptying in mice.

## INTRODUCTION

Bile acid signaling in the gastrointestinal tract is important for the integrated regulation of lipid, glucose, and energy metabolism (21). Dysregulated bile acid signaling is associated with a wide range of diseases, including cholestatic liver diseases, dyslipidemia, fatty liver diseases, cardiovascular diseases, and Type 2 diabetes (21). The G protein-coupled bile acid receptor 1, also known as TGR5 (Takeda G protein-coupled receptor 5), is a cell-surface receptor widely expressed in human tissue, including the intestine (15). In intestinal L cells, a type of enteroendocrine cell abundant in the ileum and colon, TGR5 agonists induce systemic release of glucagon-like peptides (GLPs) 1 and 2 and peptide YY (PYY) (34). GLP-1 is an incretin that has insulinotropic effects in the pancreas to regulate glucose homeostasis, as well as extra pancreatic indirect metabolic effects (6). GLP-1 analogs have been found to be useful for the treatment of patients with Type 2 diabetes (11, 13), and they are currently under investigation as a treatment for nonalcoholic steatohepatitis (NASH), a severe form of nonalcoholic fatty liver disease (NAFLD) (1, 2). NASH is histologically characterized by excessive accumulation of hepatic fat (steatosis) that is associated with hepatocyte injury and inflammation, steatohepatitis, and fibrosis (18). Both insulin resistance in the liver and adipose tissue and excessive hepatic lipogenesis contribute to NAFLD/NASH progression (4, 20, 27). Studies in animal models of NASH have shown that GLP-1 therapy improves insulin sensitivity and reduces hepatic glucose production, and can reduce hepatic steatosis, inflammation, steatohepatitis, and fibrosis (12, 25, 33). In patients with NASH, the GLP-1 analog liraglutide promotes histological resolution of the condition and reduces metabolic dysfunction, insulin resistance, and lipotoxicity (1, 2). Subcutaneous injection of GLP-2 has been found to improve intestinal epithelial barrier function (3) and accelerate liver regeneration in mice that had undergone partial hepatectomy (16).

TGR5 agonists may have therapeutically beneficial effects in patients with NAFLD/NASH via stimulation of GLP-1 and GLP-2 production (31, 35). However, clinical development of TGR5 agonists for the treatment of metabolic diseases has been hindered by side effects associated with systemic TGR5 agonism, including inhibition of gallbladder emptying (5, 22), which can lead to gallstone formation, and changes in heart rate and blood pressure (28, 29). Therapeutically targeting intestinal TGR5 with molecules that are not systemically absorbed could potentially avoid these unwanted side effects by preventing activation of TGR5 receptors expressed in tissues elsewhere in the body, such as the gallbladder. To date, studies of intestinally targeted TGR5 agonists have had mixed results in mouse models of diabetes. Although the agonists show glucose-lowering activity and have reduced gallbladder-based side effects compared with absorbed TGR5 agonists, they are still partly absorbed from the gastrointestinal tract; gallbladder-based side effects, thus, occur to some extent (8, 14, 23). Here, we characterize RDX8940 (formerly RDX009-01), a novel, orally administered TGR5 agonist designed to be minimally systemic, potent, and selective, and investigate its effects in mice fed a Western diet, a model of NAFLD, and mild insulin resistance, to determine its potential therapeutic value to patients with metabolic diseases, such as NAFLD/NASH.

## MATERIALS AND METHODS

### 

#### RDX8940 activity in vitro.

Takeda G protein-coupled receptor 5 (TGR5) agonist activities of RDX8940 and the known TGR5 agonists INT-777 and GPBAR-A were assessed in human embryonic kidney (HEK) 293 cells (American Type Culture Collection, Manassas, VA) stably transfected to heterologously express human or mouse TGR5, as described previously (9).

#### RDX8940 selectivity.

Agonist selectivity was assessed in HEK 293 cells stably transfected to heterologously express bile acid targets: the farnesoid X receptor (FXR) or the apical sodium-dependent bile acid transporter (ASBT).

A synthetic gene for human ASBT (*SLC10A2*) was made on the basis of the reference GenBank sequence BC130521 for human ASBT and optimized for expression in human cells. The *SLC10A2* gene was cloned into the pJ603 expression vector (ATUM, Newark, CA) for transient expression in HEK 293 cells. [Taurine-^3^H]-taurocholic acid was obtained from American Radiolabeled Chemicals (Saint Louis, MO). HEK 293 cells were seeded into 96-well plates at 25,000 cells/well and cultured overnight. Lipofectamine 2000 (Thermo Fisher Scientific, Waltham, MA) was used to introduce the ASBT expression construct into the cells, and the cells were allowed to approach confluence during a second overnight incubation. Medium was aspirated from the cultures, and the cells were washed once with choline uptake buffer, consisting of (in mM) 10 HEPES, 116 choline chloride, 5.3 KCl, 1.8 CaCl_2_, 11 glucose, 1.1 KH_2_PO_4_ (pH 7.4). Cells were then overlaid with either choline uptake buffer or sodium uptake buffer, consisting of (in mM) 10 HEPES, 116 sodium chloride, 5.3 KCl, 1.8 CaCl_2_, 11 glucose, 1.1 KH_2_PO_4_ (pH 7.4), containing 10 μM [^3^H]-taurocholic acid. At time intervals, the uptake solution was removed, and cells were washed twice with ice-cold choline uptake buffer to remove unincorporated radioactivity. Cells were lysed by the addition of 20 μl of 0.1% Tween 80 followed by 100 μl of scintillation fluid and counted using a TopCount Scintillation Counter (Perkin Elmer, Waltham, MA). For evaluation of RDX8940, 10 mM stock solutions of test compounds in DMSO were serially diluted threefold in DMSO. Aliquots from the resulting dilution series were further diluted 375-fold into sodium uptake buffer containing 10 µM [^3^H]-taurocholic acid to obtain eight concentrations of RDX8940, ranging from 35 nM to 30 µM. These solutions were overlaid in duplicate onto washed cells, and uptake was measured as described above following a 40-min incubation at room temperature. The amount of [^3^H]-taurocholic acid taken up by the cells was plotted against log [RDX8940], and the resulting curves were fitted to a three-parameter logistical equation using GraphPad Prism to determine the pIC_50_ (the negative log of the concentration that produced 50% inhibition [IC_50_]) values of test compounds.

The ability of test compounds to activate FXR (*NR1H4*) was measured using a cell-based assay kit obtained from Indigo Biosciences (State College, PA). Cells expressing human FXR and a FXR-responsive luciferase reporter gene were grown in duplicate in the presence of 0.4–50 µM test compound in buffer, according to the manufacturer’s instructions. Compounds and media were replaced with luciferase detection reagent, and the luminescence signal was read using a Molecular Devices Flexstation (Molecular Devices, Sunnyvale, CA). The luminescence response was plotted against log [drug], and the resulting curves were fitted to a three-parameter logistical equation using GraphPad Prism to determine pEC_50_ values and the magnitude of the response.

The binding selectivity of RDX8940 to a range of other receptors, transporters, and ion channels was evaluated using an 80-target pharmacology in vitro binding assay panel (Diversity Profile; Eurofins Cerep Panlabs, St. Charles, MO).

#### Ames test (with and without S9 fraction activation).

Genotoxic potential of RDX8940 was assessed using a microscale Ames test (24) at Eurofins Cerep Panlabs (St. Charles, MO). Wells that displayed bacteria growth caused by the reversion of a histidine mutation (judged by an OD_430_/OD_570_ ratio being greater than 1.0) were counted and recorded as positive counts. The significance of the difference in positive counts between the treatment (in the presence of test compound) and the control (in the absence of test compound) were calculated using the one-tailed Fisher’s exact test. Three significance levels were used: weak positive, if 0.01 ≤ *P* < 0.05, denoted as “+”; strong positive, if 0.001 *≤ P <* 0.01, denoted as “++”; and very strong positive, if *P <* 0.001, denoted as “+++”.

#### Human ether-à-go-go-related gene inhibition assay.

Inhibition of human ether-à-go-go-related gene (hERG) by RDX8940 was assayed by Zenas Technologies (New Orleans, LA). Currents were measured using the whole-cell variant of the patch-clamp method. Glass pipettes were pulled from borosilicate glass by a horizontal puller (Sutter Instrument, Novato, CA) and fire-polished to produce tip openings of 1 to 2 μm for K current recordings. Pipette tip resistance was ~1.0 to 2.0 mΩ when filled with K internal solution consisting of (in mM) 130.0 KCl, 1.0 MgCl_2,_ 5.0 HEPES, 5.0 EGTA, 7.0 NaCl, and 5.0 ATP-Na_2_, adjusted to a pH of 7.2 using KOH (Sigma). Bath temperature was measured by a thermistor placed near each analyzed cell and was maintained at 37 ± 1°C by a thermoelectric device (model no. 806-7243-01, Cambion/Midland Ross, Cambridge, MA). The external bath solution consisted of (in mM) 137.0 NaCl, 1.8 CaCl_2_, 10.0 HEPES, 5.0 EGTA, 4.0 KCl, 11.0 dextrose, adjusted to a pH of 7.4 with NaOH (Sigma). An Axopatch 1-B amplifier (Axon Instruments, Foster City, CA) was used for whole cell voltage clamping. Creation of voltage-clamp pulses and data acquisition were controlled by a computer running pClamp software (v. 9.2; Axon Instruments).

The ion channel blocking profile of compounds RDX8940 was characterized on hERG current recorded from a stably expressing human cell line (HEK 293). Data were presented as percent reduction of current amplitude. This was measured as current reduction after a steady-state effect had been reached in the presence of drug, relative to current amplitude before drug was introduced (control). Each cell served as its own control.

#### RDX8940 solubility in vitro.

Solubility of RDX8940 was assessed in fed-state simulated intestinal fluid (FeSSIF) and a potassium phosphate buffer pH 2.5 (150 mM ionic strength), as described previously (9).

#### RDX8940 permeability in vitro.

Permeability of RDX8940 was estimated using human colorectal adenocarcinoma (Caco-2) and Madin-Darby canine kidney (MDCK) bidirectional permeability assays. Caco-2 cells and MDCK cells were cultured into monolayers on 96-well Millicell 0.4-µm polycarbonate membrane inserts (EMD Millipore, Billerica, MA) for 21–25 days, and on HTS Transwell 24-well 0.4-µm polyester membrane inserts (Corning, NY) for 48 h, respectively, before each assay was carried out, as described previously, with the following modifications (9). RDX8940 (10 μM in transport buffer with a final DMSO concentration of 1% or 0.1% for Caco-2 or MDCK, respectively) was added to the donor side of the inserts [on either the apical (A) or basolateral (B) side of the cells] in duplicate, and transport buffer was added to the receiver side. In the MDCK assay, the P-gp inhibitor verapamil was included on the A and B sides at 100 μM with RDX8940 or control compounds to determine whether RDX8940 was potentially a P-gp substrate. Samples were taken from the donor side before incubation and the donor and receiver sides after incubation, and they were analyzed by HPLC-tandem mass spectrometry (HPLC-MS/MS).

In the Caco-2 permeability assay, the donor samples were diluted 1:5 with transport buffer. Two volumes of 1:1 acetonitrile:methanol and 0.1% formic acid were added to the donor and receiver samples, vortexed, and centrifuged. Samples were injected with a CTC HTS autosampler (Zwingen, Switzerland) connected to an Eksigent MicroLC 200 Plus HPLC and Triple Quad 5500 LC-MS/MS system (Sciex, Framingham, MA). Chromatographic separation was performed using a Kinetex C18 analytical column (30 mm × 2.1 mm ID, 2.6-μm particle; Phenomenex, Torrance, CA) with a gradient elution method. Mobile phase A consisted of 12 mM ammonium formate/6 mM formic acid/water. Mobile phase B consisted of 6 mM ammonium formate/3 mM formic acid/water in acetonitrile. The analyte was ionized using electrospray in positive ionization (ESI+) mode. The multiple reaction monitoring (MRM) transition used for RDX8940 was 764.1 > 583.0 *m/z*. In the MDCK assay the MRM transitions used for RDX8940 and IS were 763.3 > 582.2 and 329.1 > 162.0 *m/z*, respectively.

The following equations were used to calculate the *P*_app_, efflux ratio, and recovery of test compound in Microsoft Excel. Compounds with an efflux ratio > 5 (Caco-2) or > 2 (MDCK) indicate that they may be potential substrates of one or more efflux transporters. MRM peak areas were used instead of concentrations for the Caco-2 assay. For the MDCK assay, RDX8940 concentrations in study samples were interpolated from a standard curve based on the ratio of the peak area of RDX8940 for each calibration sample to the peak area of the IS at each calibration level using MassHunter Quantitative Analysis software (Agilent). For the Caco-2 assay,

$$\text{Caco-2 }P_{\text{app}}=\left({\text{V}}_{\text{R}}\times {\text{C}}_{\text{R,end}}\right)/\left[A\times \Delta t\times \left({\text{C}}_{\text{D,mid}}–{\text{C}}_{\text{R,mid}}\right)\right].$$

For the MDCK assay,

$$\text{MDCK }P_{\text{app}}=\left({\text{V}}_{\text{R}}\times {\text{C}}_{\text{R,end}}\right)/\left(A\times t\times {\text{C}}_{\text{D0}}\right).$$

The equation for the efflux ratio is

$$\text{ER}=\left(P_{\text{app}}\text{B}\to \text{A}\right)/\left(P_{\text{app}}\text{A}\to \text{B}\right),\;and$$

finally, the equation for recovery is

$$\text{Recovery}=\left[\left({\text{C}}_{\text{D,end}}\times {\text{V}}_{\text{D}}\right)+\left({\text{C}}_{\text{R,end}}\times {\text{V}}_{\text{R}}\right)\right]/\left({\text{C}}_{\text{D0}}\times {\text{V}}_{\text{D}}\right)\times 100,$$

where *P*_app_ is apparent permeability (cm/s), ER is efflux ratio, recovery is assay recovery of compound (%), V_D_ is volume of donor (ml), V_R_ is volume of receiver (ml), C_D0_ is concentration of test compound on donor side at *time 0* (μM), C_D,end_ is concentration of test compound on donor side at the end time point (μM), C_R,end_ is concentration of test compound on receiver side at the end time point (μM), C_D,mid_ is mean donor side concentration of test compound *time 0* and at end time point (μM), C_R,mid_ is one-half of the concentration of test compound on receiver side at the end time point (μM), *A* is surface area of Transwell membrane insert (cm^2^), and *t* is total incubation time (s).

#### Ethical use of animals.

All animal experiments were conducted using protocols approved by the Ardelyx Institutional Animal Care and Use Committee. All experiments were performed in accordance with the Guide for the Care and Use of Laboratory Animals from the Institute for Laboratory Animal Research (National Research Council, Washington, DC, National Academy Press, 2011).

#### Intestinal and plasma pharmacokinetics of RDX8940 and GLP-1 induction in mice.

Adult male C57BL/6 mice (Taconic, Germantown, NY) fed standard chow (Harlan Teklad 2018, 6.2% fat) were given a single oral dose of RDX8940 10 mg/kg or 30 mg/kg (*n* = 3 per time point; formulated in 2% DMSO, 10% hydroxypropyl β-cyclodextrin [HPβCD], 10 mM PBS adjusted to pH 2–2.5 with HCl), and blood samples were taken 0.5, 1, 2, and 4 h after drug administration for the measurement of plasma RDX8940 concentrations.

Stools were collected for 48 h after dosing to assess recovery of RDX8940. In a separate experiment, adult male C57BL/6 mice (Taconic) were given a single oral dose of RDX8940 (30 mg/kg) or vehicle (1 mg/kg) (*n* = 18 for each dosing set; formulated in 2% DMSO-10% HPβCD-10 mM PBS). Groups of six mice from each dosing set were euthanized using isoflurane anesthesia at 1, 4, and 8 h after drug administration. At the time of euthanasia, all mice had been fasted for 8 h. Blood samples were collected into K_3_EDTA-containing tubes (Greiner-Bio-one, Kremsmünster, Austria) and were processed to plasma for measurement of total GLP-1 (tGLP-1) concentrations using an MSD immunoassay (Meso Scale Discovery, Rockville, MD). The proximal and distal small intestine, cecum, and small and distal colon were collected from each animal into five separate tubes and frozen immediately until the day of analysis. RDX8940 was quantified in mouse plasma and tissue samples using HPLC-MS/MS, as described previously with the following modifications (9). Working solutions of standards were diluted 1:10 in pooled C57BL/6 mouse K_3_-EDTA plasma (Bioreclamation IVT, Liverpool, NY) or blank homogenized tissue matrix, which was prepared by homogenizing tissue from treatment-naïve animals (pooled C57BL/6 mouse small intestine, cecum, and colon) in 1:1 acetonitrile:water with stainless-steel balls for 5 min at room temperature. Blank plasma and tissue samples were extracted to evaluate the selectivity of the analytical method in the presence of matrix. The study tissue samples were thawed at room temperature and homogenized in the same way as the blank tissue. A subset of the tissue samples was diluted 1:5 or 1:20 in blank tissue matrix, so concentrations were within the quantitative range. Plasma samples were not diluted. Supernatants of precipitated proteins were transferred and were diluted with an equal volume of water (for plasma) or diluted 1:10 with 1:1 acetonitrile:water (for tissues). Chromatographic separation was performed using a Gemini NX-C18 reverse-phase 30 × 2 mm, 3-µm analytical column (Phenomenex) with a gradient elution method. The flow rate was 0.4 ml/min, and the gradient was as follows: 10–95% B for 0–2.5 min, 95% B for 2.5–4 min, and 10% B for 4–5 min for column equilibration. The MRM transitions used to detect RDX8940 and the IS were 763.3 > 582.2 and 329.1 > 162 *m/z*, respectively. RDX8940 concentrations in the study samples were interpolated from a calibration curve based on the peak area ratio of NTX8940/IS and the theoretical concentration of each standard using MassHunter software.

#### Effects of RDX8940 on secretion of intestinal L-cell hormones in mice fed a standard diet.

Adult male C57BL/6 mice (Taconic) fed standard chow (Harlan Teklad 2018) were dosed orally with vehicle (2% DMSO, 10% HPβCD, 10 mM PBS-HCl) or RDX8940 in separate experiments, as described below. After dosing, mice were anesthetized, and blood was collected via cardiac puncture into K_3_EDTA-containing tubes (Greiner Bio-one); then, the mice were euthanized. If active GLP-1 (aGLP-1) concentrations were measured, sitagliptin and aprotinin were added to the tubes before blood collection. Blood was processed to plasma for the measurement of incretins; aGLP-1, tGLP-1, and PYY concentrations were assessed using MSD immunoassays (Meso Scale Discovery), whereas total GLP-2 (tGLP-2) concentrations were quantified using a mouse GLP-2 ELISA (ALPCO, Salem, NH).

In the first experiment, mice were administered vehicle or a single dose of RDX8940 (10, 30, or 100 mg/kg), fasted, and plasma tGLP-1 concentrations were measured 8 h after dose. In a second experiment, mice were administered repeated doses of vehicle or RDX8940 30 mg/kg twice daily over 4 days. After the last dose, the mice were fasted and plasma tGLP-1 concentrations were measured 8 h later. In a third experiment, mice were administered single or repeated doses of RDX8940 30 mg/kg twice daily over 4 days or were left untreated (*n* = 6 per group). After the single or last dose, the mice were fasted, and plasma concentrations of aGLP-1, tGLP-1, tGLP-2, and PYY were measured 8, 10, and 12 h later.

To assess the effect of coadministration of dipeptidyl peptidase-4 (DPP4) inhibitors with RDX8940, mice were administered linagliptin (10 mg/kg once daily), sitagliptin (3.6 g/l administered in drinking water), alogliptin (30 mg/kg twice daily), or vehicle (administered in drinking water) for 4 days (*n* = 8 per group). On the fourth day, the mice were given a single oral dose of RDX8940 (10, 30, or 100 mg/kg) or vehicle (2% DMSO, 10% HPβCD, 10 mM PBS-HCl), fasted, and plasma aGLP concentrations were measured 8 h later. In a separate experiment, mice were administered repeated doses of RDX8940 30 mg/kg orally twice daily with or without linagliptin 10 mg/kg orally once daily, or linagliptin or vehicle alone, for 4 days (*n* = 8 per group). After the final dose, the mice were fasted (8 h for 4-, 8-, and 12-h post-dose time points and 12 h for the 16-h post-dose time point) and were then euthanized for measurement of plasma aGLP-1 concentrations.

For assessment of aGLP-1 and tGLP-1 levels in mouse portal venous and systemic circulations, mice were administered RDX8940 30 mg/kg orally twice daily, with or without linagliptin 10 mg/kg orally once daily, or linagliptin or vehicle alone, for 4 days (repeated dosing; *n* = 8–12 per group). On the fourth day, mice were dosed once orally, fasted for 8 h, and then euthanized for measurement of plasma aGLP-1 and tGLP-1 concentrations sampled from the portal vein or by cardiac puncture.

#### Effects of RDX8940 on secretion of aGLP-1, insulin sensitivity, and liver steatosis in mice fed a Western diet.

Six-week-old male C57BL/6 mice (Taconic) were fed Western diet chow (Harlan Teklad TD.88137, 21.2% fat, 0.2% cholesterol, 42.0% kcal from fat) for 10 wk to model NAFLD and mild insulin resistance. The mice were then given RDX8940 30 mg/kg orally twice daily and/or linagliptin 10 mg/kg orally once daily or twice daily subcutaneous injections of liraglutide 400 µg (H-6724.0005; Bachem Americas, Torrance, CA) (reconstituted in sterile water) or vehicle (2% DMSO, 10% HPβCD, 10 mM PBS-HCl) orally twice daily for 4 wk (*n* = 9–10 per group). An additional vehicle (administered orally twice daily) control group (lean) was fed standard chow (Harlan Teklad 2018) throughout the study (*n* = 10). On *days 14* and *29*, mice were fasted for 4 h after the first dose, and blood was then collected via the tail vein for measurement of plasma glucose and insulin concentrations using a glucometer (Abbott Animal Health, Abbott Park, IL) and an MSD immunoassay (Meso Scale Discovery), respectively. On *day 29*, mice were fasted longer (a total of 7–8 h), were anesthetized, and had blood collected via cardiac puncture for the measurement of GLP-1 levels. Mice were then euthanized under anesthesia for the collection of liver tissue. Hepatic triglyceride and cholesterol content were determined at the University of California, Davis, CA, using the Folch method (17).

#### Effects of RDX8940 on gallbladder emptying in mice.

Adult female CD-1 mice (Charles River, Wilmington, MA; *n* = 6 per group) fed standard chow (Harlan Teklad 2018) were fasted overnight and then dosed once orally with RDX8940 (30 and 100 mg/kg) and INT-777 30 mg/kg (a systemic TGR5 agonist) formulated in vehicle (0.1% Tween 80, 10% HPβCD/HCl) or vehicle alone; they were then fed with saline or egg yolk after 1 h 45 min. Mice were euthanized under anesthesia after a further 15 min, and the gallbladders were removed and weighed.

#### Toxicology profiling of RDX8940 in mice.

Adult CD-1 mice (Charles River) fed standard rodent chow (Harlan Teklad 2018) were dosed orally by gavage once daily with RDX8940 30, 300, or 1,000 mg/kg (*n* = 6 per dose level: 3 female; 3 male) or vehicle (hydrochloric acid in deionized water, pH 2.0–2.5, 10 ml/kg) [*n* = 6: 3 female; 3 male] for 7 consecutive days to assess the tolerability of RDX8940. All animals were observed twice daily for mortality and moribundity. Clinical observations were performed and recorded daily before dosing and again 1–2 h after dosing. Individual body weights were measured daily during the dosing period. Complete necropsies were conducted on all toxicology group animals, and organ weights and macroscopic observations were recorded. Selected tissues were examined microscopically.

#### Effects of GLP-1 and GLP-2 in mice fed a diet high in fat, cholesterol and carbohydrate.

Five-week-old male C57BL/6 mice (Taconic) were fed normal chow (Harlan Teklad 2018) and given drinking water with no additives (*n* = 10) or fed a diet high in fat, cholesterol, and carbohydrate [HFCD; Harlan Teklad TD.130885, 20% protein, 43% carbohydrate, 23% fat, 0.2% cholesterol (6.6% trans fat)] and given drinking water containing 55:45 fructose: dextrose 42 g/l (*n* = 30). At 19 wk old, mice began treatment. Normal chow-fed mice and one group of HFCD-fed mice were dosed with vehicle (0.1% Tween 80, 2.5% HPβCD), and two other HFCD-fed groups were injected subcutaneously twice daily with 200 µg/kg liraglutide (Bachem Americas) or 400 µg/kg of the GLP-2 analog teduglutide (Creative Dynamics, Shirley, NY). Mice were weighed each week. Before treatment and on *days 14* and *28* of treatment, the mice were fasted for 4 h, and blood was then collected via the tail vein and processed to plasma for measurement of alanine aminotransferase (ALT), aspartate aminotransferase (AST), alkaline phosphatase (AP), glucose, triglycerides, and total cholesterol using an ACE Axcel clinical analyzer (Alfa Wassermann, West Caldwell, NJ). After 34 days of treatment, the mice were fasted for 6–8 h and were euthanized under anesthesia. Blood was collected and processed to plasma for measurement of insulin [using an MSD immunoassay (Meso Scale Discovery)], and ALT, AST, AP, and total bilirubin levels [using an ACE Axcel clinical analyzer (Alfa Wassermann)]. The entire liver was collected and weighed, and samples of liver were weighed for measurement of triglycerides and total cholesterol levels at University of California, Davis, CA [using the Folch method (17)]; histological assessments of steatosis, inflammation, and fibrosis were performed using hematoxylin-and-eosin stains, and Sirius red stains (Wax-it Histology Services, Vancouver, BC, Canada and Pacific Tox Path, Ellensburg, WA). Scoring of hepatic pathology was conducted according to descriptions provided by Thoolen et al. (32). For gene expression studies, total RNA from mouse liver tissues was isolated using a RNeasy 96 universal tissue kit (Qiagen, Hilden, Germany). A gene expression library was prepared with 100 ng of each RNA sample using a TruSeq Stranded mRNA Library NeoPrep kit (Illumina, Hayward, CA). Library samples were subsequently multiplexed and sequenced using an Illumina NextSeq 500 sequencer (San Diego, CA). Sequences of two 75-base pair ends were obtained for all samples and mapped to the mouse transcriptome, using STAR (Spliced Transcripts Alignment to a Reference) alignment with default parameters (Basespace Cloud, Illumina) and normalized using trimmed mean of M-values method in EdgeR software (Bioconductor; https://www.bioconductor.org/). Differentiation gene expression analysis was evaluated using EdgeR. R platform (https://www.r-project.org/) was used for principal component analysis and heat map analysis. Gene ontology and network correlation analysis were performed using Gorilla (http://cbl-gorilla.cs.technion.ac.il/) and WGNCA (https://horvath.genetics.ucla.edu/html/CoexpressionNetwork/Rpackages/WGCNA/).

#### Statistical analyses.

End points with test agents were compared using one- or two-way ANOVA with Holm–Šídák’s test. Parametric left-censored data and nonparametric data were analyzed using the Kruskal-Wallis test followed by Dunn’s test.

## RESULTS

### 

#### RDX8940 activity, selectivity, and toxicity.

Results of cellular assays indicated that RDX8940 had superior activity to known TGR5 agonists (Table 1). RDX8940 was more than 4,400-fold more selective for TGR5 than for the other bile acid targets FXR [concentration that produces 50% of the maximum effect (EC_50_) < 10 μM) and ASBT [concentration that produces 50% inhibition (IC_50_) < 30 μM). In addition, RDX8940 (10 μM) bound only two targets to an extent greater than 80% in an 80-target in vitro pharmacology panel; neither of these targets is exposed to the gut lumen. No potential for mutagenicity was seen with RDX8940 (up to 100 μM) in the Ames test (with or without S9 fraction activation). Potential therapeutic compounds that inhibit hERG, a potassium channel anti-target, have the propensity to cause cardiac arrhythmia (30), but no hERG potassium channel liability was observed with RDX8940 (<5% inhibition at 30 μM, manual patch clamp).

**Table 1.** Potency of RDX8940 compared with other TGR5 agonists

	Cellular TGR5 cAMP EC_50_	
Agonist	EC_50_, nM	Efficacy, %
Mouse		
INT-777	2,500	92
GPBAR-A	50	100
RDX8940	0.25	100
Human		
INT-777	10,000	104
GPBAR-A	130	101
RDX8940	2.5	92

EC_50_, concentration that produces 50% of the maximum effect; TGR5, G protein-coupled bile acid receptor.

#### RDX8940 solubility and permeability.

RDX8940 (1 mg/ml nominal) was highly soluble in FeSSIF pH 6.5 and simulated gastric fluid pH 2.5 (0.99 mg/ml and 0.98 mg/ml, respectively). The apparent permeability coefficient (*P*_app_) of RDX8940 was low across monolayers of both Caco-2 and MDCK cells from the A to the B side (Caco-2, 0.01 × 10^−6^ cm/s; MDCK, 0.56 × 10^−6^ cm/s) and the B to the A side (Caco-2, 0.84 × 10^−6^ cm/s; MDCK, 0.40 × 10^−6^ cm/s; Table 2). The efflux ratio of RDX8940 across MDCK cells was low (0.7), indicating that RDX8940 was not effluxed. Although the efflux ratio of RDX8940 across Caco-2 cells was high (84.0), suggesting that RDX8940 was effluxed, this result was inconclusive owing to the low permeability of RDX8940 (*P*_app_ < 1 × 10^−6^ cm/s) in the B to A direction. The recovery of RDX8940 was high across MDCK cells (A to B, 99%; B to A, 95%) but low across Caco-2 cells (A to B, 45%; B to A, 36%). As expected, the P-gp inhibitor, verapamil, had no effect on the permeability of RDX8940 across MDCK cells in either direction, probably because of the low *P*_app_ observed in the B to A direction without verapamil. These molecular properties of RDX8940 are consistent with those found for minimally absorbed compounds.

**Table 2.** Permeability of RDX8940

Permeability	*P*_app_ A→B,cm/s × 10^−6^	*P*_app_ B→A,cm/s × 10^−6^	Efflux Ratio, B→A:A→B	Recovery,A→B, %	Recovery,B→A, %
MDCK					
RDX8940	0.56	0.40	0.72	NC	NC
Atenolol	0.92	0.64	0.70	NC	NC
Colchicine	0.85	2.31	2.72	NC	NC
Propanolol	14.19	7.03	0.50	NC	NC
RDX8940 + verapamil	0.53	0.36	0.68	99	95
Atenolol + verapamil	0.70	0.58	0.82	113	106
Colchicine + verapamil	1.04	1.27	1.22	120	113
Propranolol + verapamil	13.07	8.71	0.67	89	98
Caco-2					
RDX8940	0.01	0.84	84.00	45	36
Colchicine	0.23	6.18	27.44	69	80
Labetalol	19.45	31.61	1.63	64	80
Propanolol	67.06	19.62	0.29	56	78
Ranitidine	0.91	3.02	3.34	78	87

RDX8940 chemical descriptors: molecular weight, > 700 g/mol; LogP, 2.28 ± 0.12; PSA, > 200 Å². A, apical; B, basolateral; LogP, log partition coefficient; MDCK, Madin-Darby canine kidney; MW, molecular weight; NC, not calculated due to a donor-sampling error at the end time; *P*_app_, apparent permeability coefficient; PSA, polar surface area.

#### Intestinal and plasma pharmacokinetics of RDX8940 and GLP-1 induction in mice.

**Fig. 1.**
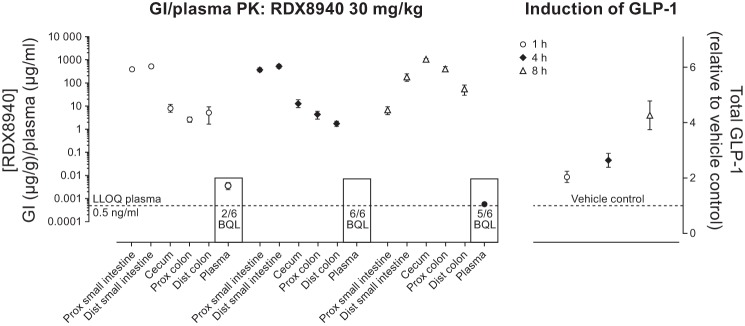
Intestinal and plasma PK of RDX8940 and glucagon-like peptide-1 (GLP-1) induction in mice at 1, 4, and 8 h after a single oral dose of RDX8940 30 mg**/**kg. Data are expressed as means ± SE (*n* = 6 for each dosing set at each time point). BQL, below quantification level; Dist, distal; GI, gastrointestinal; GLP-1, glucagon-like peptide-1; LLOQ, lower limit of quantification; PK, pharmacokinetics; Prox, proximal.

RDX8940 exposure in mice was low (area under the plasma concentration-time curves <2–21 ng·ml^−1^·h^−1^) and 80–86% of the dose (10 and 30 mg/kg) was recovered in stools (data not shown). After a single dose of RDX8940 (30 mg/kg), plasma concentrations of RDX8940 were below the quantification level (or BQL; 0.5 ng/ml) for two of six, six of six, and five of six animals after 1, 4, and 8 h, respectively (Fig. 1). Quantifiable plasma concentrations of RDX8940 after 1 and 8 h were 3.7 ng/ml (mean of 4 animals) and 0.6 ng/ml, respectively. Throughout the intestine, RDX8940 concentrations were relatively high; highest levels were observed in the proximal small intestine (mean ± SD 380 ± 65 and 365 ± 131 mg/g) and distal small intestine (522 ± 220 and 516 ± 213 mg/g) at 1 and 4 h, respectively. At 8 h, RDX8940 concentrations were highest in the distal small intestine, cecum, and proximal and distal colon (185 ± 172, 1,078 ± 84, 397 ± 170, and 55 ± 63 mg/g, respectively). Plasma concentrations of tGLP-1 increased over time (2.1 ± 0.5-, 2.7 ± 0.6-, and 4.3 ± 1.3-fold above vehicle control at 1, 4, and 8 h, respectively). Peak tGLP-1 plasma levels, therefore, occurred while RDX8940 concentrations were high in the distal small intestine and further along the gastrointestinal tract 6 h after RDX8940 concentrations were at peak levels in plasma.

#### Effects of RDX8940 on secretion of intestinal L-cell hormones in mice fed a standard diet.

**Fig. 2.**
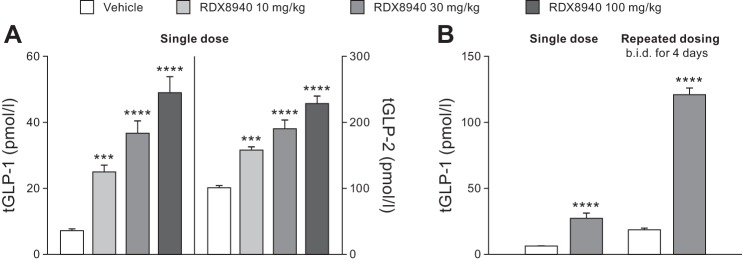
RDX8940 induces dose-dependent increases in total glucagon-like peptide-1 (tGLP-1) and tGLP-2 secretion in mice fed a standard diet (*A*), which is enhanced by repeated dosing (*B*). Bars show mean values; error bars show standard error of the mean (*n* = 8 per group). tGLP-1 and tGLP-2 levels were measured 8 h after the last dose. The single-dose data presented in *B* are the same as those shown in *A*. ***P < 0.001 and ****P < 0.0001 vs. vehicle. b.i.d., twice daily.

**Fig. 3.**
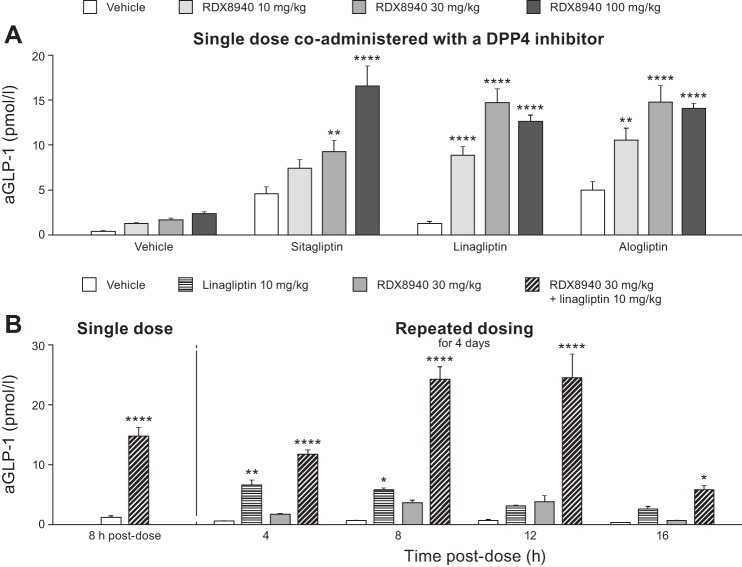
RDX8940-induced increase in plasma active glucagon-like peptide-1 (aGLP-1) in mice fed a standard diet is enhanced by coadministration of dipeptidyl peptidase-4 (DPP4) inhibitors (*A*) and repeated dosing of RDX8940 (*B*). Bars show mean values; error bars show standard error of the mean (*n* = 8 per group). *A*: sitagliptin 3.6 g/l administered in drinking water, linagliptin 10 mg·kg^−1^·day^−1^ administered orally, and alogliptin 30 mg/kg twice a day administered orally. Vehicle or gliptins were administered to mice for 4 days; on the fourth day, the mice were fasted and were then dosed once orally with RDX8940; aGLP-1 was measured 8 h later. *B*, *left*: single-dose data that was presented in *A*. Repeated dosing was carried out by administering test agents twice a day (linagliptin once/day) orally to mice for 4 days. After the final dose, the mice were fasted (8 h for 4-, 8-, and 12-h post-dose time points and 12 h for the 16-h post-dose time point), and aGLP-1 was measured at the indicated time points. *P < 0.05, **P < 0.01, and ****P < 0.0001 vs. vehicle. aGLP-1, active glucagon-like peptide-1; DPP4, dipeptidyl peptidase-4.

**Fig. 4.**
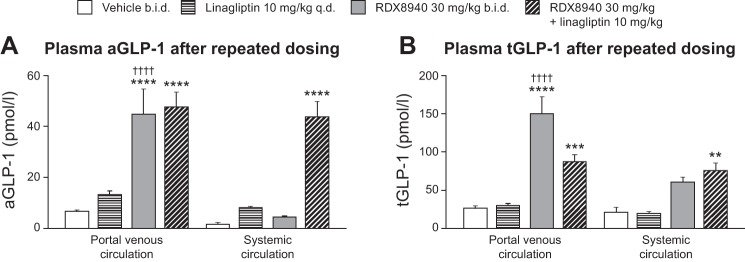
Portal venous levels of aGLP-1 (*A*) and tGLP-1 (*B*) in mice fed a standard diet are increased by RDX8940 alone, whereas coadministration with a dipeptidyl peptidase-4 inhibitor is required to increase systemic levels of aGLP-1 and tGLP-1. Bars show mean values; error bars show standard error of the mean (*n* = 8–12 per group). Test agents were administered twice daily orally to mice for 4 days. On the fourth day, the mice were fasted and were dosed once orally; aGLP-1 and tGLP-1 levels were measured 8 h later. **P < 0.01, ***P < 0.001, and ****P < 0.0001 vs. vehicle; ^††††^*P* < 0.0001 vs. systemic circulation. aGLP-1, active glucagon-like peptide-1; b.i.d., twice daily; GLP-1, glucagon-like peptide-1; tGLP-1, total glucagon-like peptide-1.

RDX8940 induced dose-dependent increases in tGLP-1 and tGLP-2 secretion (Fig. 2*A*; Table 3). Repeated dosing of RDX8940 increased tGLP-1 levels to a much greater extent than a single dose at 8 h after the single or final dose (Fig. 2*B*). To understand this effect better, a time course study of incretin secretion in response to single or repeat dosing of RDX8940 was performed. Single or repeat dosing of RDX8940 30 mg/kg induced elevated and sustained plasma levels of aGLP-1, tGLP-1, tGLP-2, and PYY compared with untreated control (Table 3). Peak levels of these hormones were higher after 4 days of twice-daily RDX8940 dosing than after a single dose and occurred earlier (Table 3). At 8 h after dosing, single doses of RDX8940 induced greater increases in plasma aGLP-1 levels when administered in combination with different DPP4 inhibitors, which slow aGLP-1 degradation, than when administered alone (Fig. 3*A*). After repeat dosing, RDX8940 plus linagliptin again induced a substantially greater mean increase in plasma aGLP-1 levels than RDX8940 alone at 8 h after the final dose; however, the mean increase was approximately twice that seen with a single dose of the combination at this time point (Fig. 3*B*). After repeat dosing, aGLP-1 levels in the portal vein were significantly higher with RDX8940 treatment than with vehicle (*P* < 0.01), whereas treatment with linagliptin did not significantly affect portal aGLP-1 levels (*P* = 0.91), and there was no further increase in portal aGLP-1 when linagliptin was added to RD8940 treatment (*P* = 0.99; Fig. 4*A*). The lack of effect of linagliptin alone or in combination with RDX8940 on portal aGLP-1 levels indicates that intestinally derived aGLP-1 is not subject to extensive DPP4-mediated degradation before accessing the liver. Monotherapy treatment with RDX8940 (*P* = 0.92) or linagliptin (*P* = 0.48) did not significantly increase systemic concentrations of aGLP-1; however, the combination of RDX8940 and linagliptin treatment did significantly increase systemic aGLP-1 levels (*P* < 0.001; Fig. 4*A*), indicating extensive systemic DPP4-mediated inactivation of aGLP-1.

**Table 3.** Effects of single and repeated dosing with RDX8940 on systemic secretion of intestinal L-cell hormones in mice fed a standard diet

		RDX8940-Induced Fold Change in Hormone Levels*^a^*	
Hormone	Time After Single/Last Dose, h	Single Dose, 30 mg/kg	Repeated Dosing, 30 mg/kg Twice Daily for 4 days
aGLP-1	8	9.1	27.0
	10	12.9	15.3
	12	16.5	12.8
tGLP-1	8	6.3	20.7
	10	10.0	9.6
	12	10.2	8.6
tGLP-2	8	2.1	4.3
	10	3.1	2.4
	12	2.4	2.4
PYY	8	8.4	20.0
	10	10.2	13.0
	12	14.3	10.0

aGLP, active glucagon-like peptide; PYY, peptide YY; tGLP, total glucagon-like peptide. *^a^*Fold change values are relative to vehicle control (*n* = 8 per group).

#### Effects of RDX8940 on secretion of aGLP-1, insulin sensitivity, and liver steatosis in mice fed a Western diet.

**Fig. 5.**
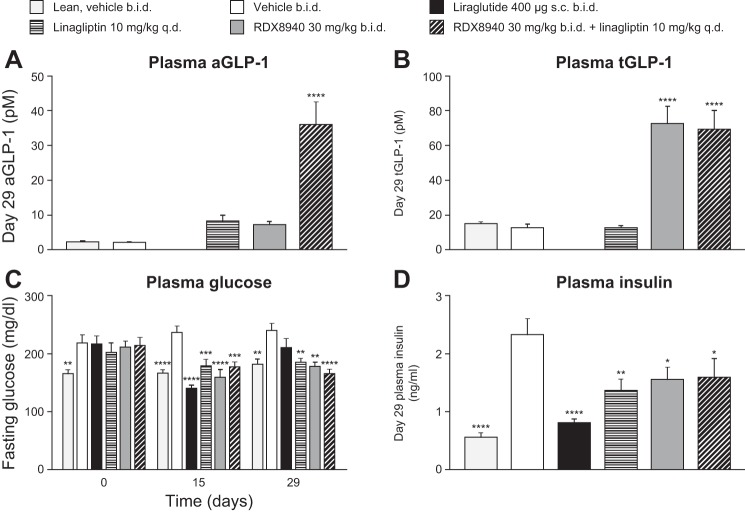
RDX8940 acts synergistically with a dipeptidyl peptidase-4 inhibitor to increase plasma levels of aGLP-1 (*A*) and tGLP-1 (*B*) in mice fed a Western diet (nonalcoholic fatty liver disease model), improving glycemic control (*C*) and insulin sensitivity (*D*). Bars show mean values; error bars show standard error of the mean (*n* = 9 or 10 per group). All mice were fed a Western diet except for the lean group. End points were measured in fasted mice 7–8 h post-dose on *day 29* of treatment unless otherwise stated. *P < 0.05, **P < 0.01, ***P < 0.001, and ****P < 0.0001 vs. Western diet vehicle. aGLP-1, active glucagon-like peptide-1; b.i.d., twice daily; q.d., once daily; sc, subcutaneous; tGLP-1, total glucagon-like peptide-1.

**Fig. 6.**
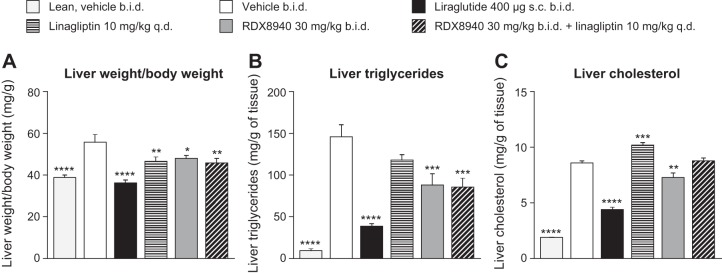
RDX8940 improves measures of liver steatosis [liver weight/body weight (*A*), liver triglycerides (*B*), and liver cholesterol (*C*)] in mice fed a Western diet (nonalcoholic fatty liver disease model). Bars show mean values; error bars show standard error of the mean (*n* = 9 or 10 per group). Values in parentheses are doses in milligrams unless stated otherwise. All mice were fed a Western diet except for those in the lean group, which were fed standard chow. End points were measured in fasted mice 7–8 h post-dose on *day 29* of treatment. *P < 0.05, **P < 0.01, ***P < 0.001, and ****P < 0.0001 vs. Western diet vehicle. b.i.d., twice daily; q.d., once daily; sc, subcutaneous.

After 4 wk of RDX8940 treatment in mice modeling NAFLD, plasma tGLP-1 levels had increased to a greater extent than aGLP-1 levels, which increased significantly only when RDX8940 was administered in combination with linagliptin (Fig. 5, *A* and *B*). RDX8940, with or without linagliptin, lowered 4-h fasted plasma glucose (*days 15* and *29*) and insulin (*day 29*) levels compared with vehicle, as did both linagliptin alone and the GLP-1 analog liraglutide (Fig. 5, *C* and *D*). Like liraglutide, RDX8940 administered alone reduced liver weight (*P* < 0.05) and hepatic triglyceride (*P* < 0.001) and cholesterol (*P* < 0.01) levels (Fig. 6).

#### Effects of RDX8940 on gallbladder emptying in mice.

**Fig. 7.**
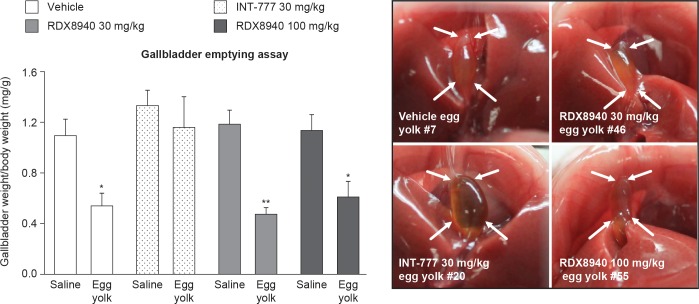
RDX8940 does not inhibit gallbladder emptying. Bars show mean values; error bars show standard error of the mean (*n* = 6 per group). Images from mice fed egg yolk show inhibition of gallbladder emptying (see arrows) following dosing with systemic TGR5 agonist INT-777 at 30 mg/kg, but not with RDX8940 30 mg/kg or 100 mg/kg. *P < 0.05 and **P < 0.01 vs. saline.

RDX8940, like vehicle but unlike the systemic TGR5 agonist INT-777, significantly reduced gallbladder weight in mice fed egg yolk compared with those fed saline (*P* < 0.05), indicating that RDX8940 did not inhibit gallbladder emptying (Fig. 7).

#### Toxicology profiling of RDX8940 in mice.

**Fig. 8.**
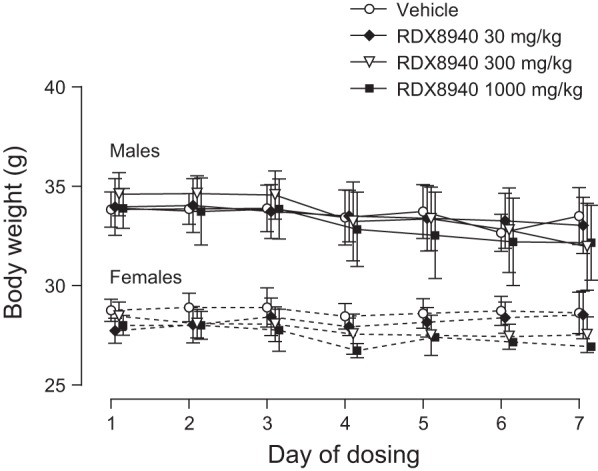
Oral once-daily RDX8940 administration over seven consecutive days has no effect on body weight of mice. Data shown are mean values; error bars show standard error of the mean (*n* = 6 per group).

Oral administration of RDX8940 to CD-1 mice at dose levels of 30, 300, and 1,000 mg·kg^−1^·day^−1^ for 7 consecutive days was tolerated at all dosage levels in the toxicology group animals. There were no test article-related clinical observations, and there was no significant effect of RDX8940 administration on body weight at any dose level relative to vehicle control (Fig. 8). Review of the gross necropsy revealed no observations that were considered to be test article-related at any of the dose levels. Test article-related microscopic findings were restricted to the observation of a mild-to-moderate reduction in lymphocyte cellularity in the thymus at the 1,000 mg/kg dose in both males and females, accompanied by a reduction in thymus weight of ~25%. Therefore, the no-observed, adverse-effect level of RDX8940 in the 7-day dose-range-finding study was determined to be 300 mg·kg^−1^·day^−1^, which produced no significant effects on organ weights or histological findings. Pathologies of known TGR5 agonists (19, 28, 29), seen in preclinical animal studies, including cardiac, hepatic, and pancreatic toxicities, were not observed.

#### Effects of GLP-1 and GLP-2 agonists in mice fed an HFCD.

**Fig. 9.**
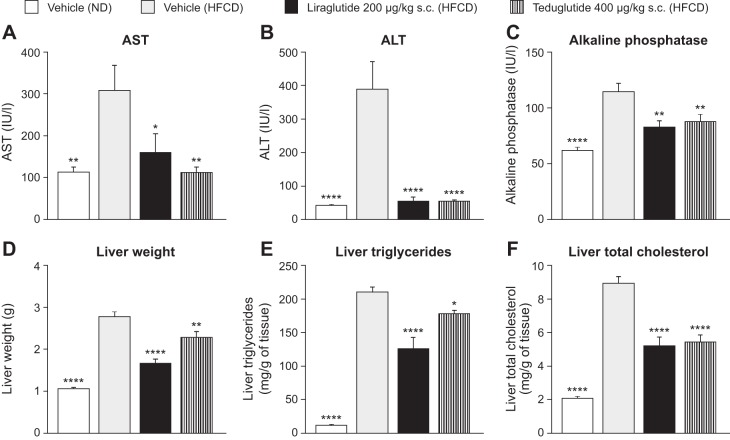
The GLP-1 agonist liraglutide and the GLP-2 agonist teduglutide reduced hepatic analyte levels (*A–C)* and improved hepatic characteristics (*D–F*) in mice fed an HFCD (a model of nonalcoholic fatty liver disease and mild Type 2 diabetes). Data are expressed as means ± SE (*n* = 10 per group). Statistical analyses were performed using one-way ANOVA followed by Holm-Šídák’s multiple-comparisons test. *P < 0.05, **P < 0.01, and ****P < 0.0001 vs. vehicle-HFCD-fed group. ALT, alanine aminotransferase; AST, aspartate aminotransferase; GLP, glucagon-like peptide; HFCD, high fat, cholesterol, and carbohydrate diet; IU, international units; ND, normal diet; sc, subcutaneous.

**Fig. 10.**
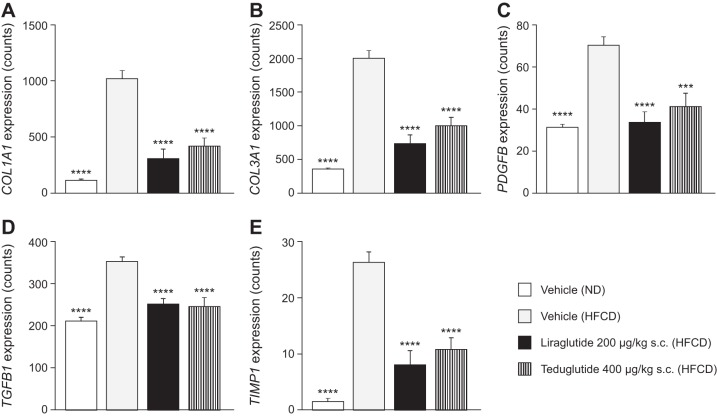
*A–E*: GLP-1 agonist liraglutide and the GLP-2 agonist teduglutide abrogated the increased expression of many hepatic genes in mice fed an HFCD (a model of nonalcoholic fatty liver disease and mild Type 2 diabetes). Data are expressed as means ± SE (*n* = 10 per group). Statistical analyses were performed using one-way ANOVA followed by Holm–Šídák’s multiple-comparisons test. ***P < 0.001 and ****P < 0.0001 vs. vehicle-HFCD-fed group. COL1A1, collagen type 1 alpha 1; COL3a1, collagen type 3 alpha 1; GLP, glucagon-like peptide; HFCD, high fat, cholesterol, and carbohydrate diet; ND, normal diet; PDGFB, Platelet-derived growth factor subunit B; sc, subcutaneous; TGFB1, transforming growth factor beta 1; TIMP1, tissue inhibitor of metalloproteinase 1.

As a supplement to the investigation of TGR5 agonism by RDX8940 in mice fed a Western diet, the effects of direct GLP-1 and GLP-2 agonism on hepatic analytes and characteristics in mice fed an HFCD (a model of NAFLD and mild Type 2 diabetes) were evaluated, using the GLP-2 agonist, teduglutide, and the GLP-1 agonist, liraglutide. As expected, compared with vehicle-treated mice fed a normal diet, vehicle-treated HFCD-fed mice had significant increases in the following: body weight; liver weight; macrovesicular and microvesicular steatosis scores; triglyceride, total cholesterol, and TNF-α content; and plasma glucose, total cholesterol, ALT, AST, and AP levels; as well as decreased plasma triglyceride levels (Table 4; Fig. 9). In HFCD-fed mice, liraglutide and teduglutide both significantly decreased hepatic weight, triglyceride and total cholesterol content, and macrovesicular steatosis scores, as well as plasma ALT, AST, and AP levels, compared with vehicle (Table 4; Fig. 9). Liraglutide also significantly decreased body weight (*day 33*); 4-h fasted plasma glucose concentration, triglyceride, and total cholesterol levels (*day 28*); insulin levels (*day 34*); hepatic microvesicular steatosis scores; and TNF-α content in HFCD-fed mice compared with vehicle, whereas teduglutide treatment had no significant effects on any of these end points. Neither liraglutide nor teduglutide significantly affected plasma total bilirubin levels (data not shown) or hepatic fibrosis scores. In HFCD-fed mice, the expression of many genes involved in inflammation and fibrotic processes in the liver were increased; liraglutide and teduglutide treatment abrogated this effect in many cases (Fig. 10).

**Table 4.** Effects of the GLP-1 agonist liraglutide and the GLP-2 agonist teduglutide in mice fed an HFCD

	Normal Diet	HFCD		
Analyte	Vehicle twice daily	Vehicle twice daily	Liraglutide	Teduglutide
*n*	10	10	10	10
Body weight, g	28.7 ± 0.8****	36.0 ± 0.4	31.4 ± 0.8***	37.9 ± 0.9
Plasma glucose, mg/dl	190 ± 8*	220 ± 7	185 ± 13*	205 ± 5
Plasma insulin, ng/ml	0.76 ± 0.29	1.89 ± 0.54	0.40 ± 0.11*	0.95 ± 0.20
Plasma total cholesterol, mg/dl	141 ± 6****	297 ± 8	230 ± 16 **	279 ± 16
Plasma triglycerides, mg/dl	105 ± 3***	88 ± 3	59 ± 3****	84 ± 1
Hepatic TNFα content, pg/ml*^a^*	2.50***	0.58	2.50**	3.50
Macrovesicular steatosis score*^a^*	0.00****	4.00	1.75**	2.00*
Microvesicular steatosis score*^a^*	0.0****	4.0	0.5****	2.0
Fibrosis score*^a^*	0	2	2	2

Values are means ± SE unless stated otherwise. GLP, glucagon-like peptide; HFCD, high fat, cholesterol, and carbohydrate diet (a model of nonalcoholic fatty liver disease and mild Type 2 diabetes).

a Values are medians. For parametric data, statistical analyses were performed using one-way ANOVA followed by Holm-Šídák’s multiple-comparisons test. Parametric left-censored data and nonparametric data were analyzed using the Kruskal-Wallis test followed by Dunn’s test.

* *P* < 0.05,

** *P* < 0.01,

*** *P* < 0.001, and

**** *P* < 0.0001 vs. vehicle-HFCD-fed group.

## DISCUSSION

TGR5 agonists have the potential to be an effective treatment for metabolic diseases, such as NAFLD/NASH, owing to their ability to lower glucose-stimulated insulin release, slow gastric emptying, and decrease food intake via stimulation of GLP-1 release, and improve intestinal barrier function, thereby lowering whole body inflammation, via GLP-2 production (3, 5, 8, 22, 31, 35). However, their use has until now been associated with undesirable side effects, such as excessive gallbladder filling and blockade of gallbladder emptying, caused by systemic TGR5 agonism in off-target tissues (5, 8, 14, 20, 23, 25). Here, we have shown that RDX8940 is a potent, selective, and minimally systemic oral TGR5 agonist that induces GLP-1, GLP-2, and PYY secretion from mouse intestinal L cells and improves liver steatosis and insulin sensitivity in a model of NAFLD and mild insulin resistance. Furthermore, we have shown that RDX8940 does not inhibit gallbladder emptying in mice.

RDX8940 had superior activity to known TGR5 agonists, was more selective for TGR5 than for other bile acid targets, did not bind to pharmacological targets that are exposed to the intestinal lumen, and displayed no potential for mutagenicity. RDX8940 was highly soluble in simulated intestinal fluid and showed low permeability across MDCK and Caco-2 cell monolayers, consistent with findings with minimally absorbed compounds. Recovery of RDX8940 across cell monolayers was high in the MDCK assay but low in the Caco-2 assay, potentially because of insolubility over time during the latter assay, metabolism, or nonspecific binding to Caco-2 cells or the assay plate. RDX8940 efflux findings were inconclusive, and additional studies with P-gp or breast cancer resistance protein inhibitors could be used to confirm whether RDX8940 is an efflux transporter substrate. However, on the basis of the permeability data presented here, apparent permeability from the A to the B direction is not expected to increase significantly in the presence of inhibitors, so the permeability classification is unlikely to change.

Exposure of RDX8940 in mouse plasma was low and a high proportion of RDX8940 was recovered in stools. Levels of tGLP-1 peaked 8 h after dosing; at this time point, RDX8940 was barely detectable in plasma, but its concentrations were at their highest in the distal small intestine and proximal colon regions of the intestine, where there is an abundance of L cells. This suggests that RDX8940 induces GLP-1 secretion from the apical side of the intestine and not via systemic exposure at the basolateral surface of the intestine.

RDX8940-induced increases in plasma aGLP-1 levels in mice were enhanced by repeated dosing and by coadministration of DPP4 inhibitors. This observation may be explained, in part, by the finding by Morimoto et al. (26) that protracted activation of TGR5 in mice results in upregulation of the prohormone convertase 1/3, an enzyme that mediates processing of proglucagon to aGLP-1. Elucidation of the precise mechanism by which aGLP-1 secretion is enhanced by repeated dosing of RDX8940 will require further investigation. RDX8940 increased hepatic exposure to aGLP-1, demonstrated by elevated aGLP-1 levels in the portal vein, without requiring coadministration of a DPP4 inhibitor. Portal venous and local gastrointestinal concentrations of incretins elicited by intestinal secretion have previously been studied by D’Alessio et al. (10), who observed that GLP-1 was disproportionately transported in intestinal lymph relative to both portal and peripheral plasma in rats after liquid meal instillation to the gastrointestinal tract. Our findings are most likely attributable to the action of RDX8940 in the colon, which results in extended exposure of the liver and gastrointestinal mucosa to aGLP1 concentrations in considerable excess of those observed in the systemic circulation.

In mice fed a Western diet, RDX8940, like liraglutide, improved insulin sensitivity and measures of liver steatosis, in line with the previously reported effects of GLP-1 therapy in animal models of NASH (12, 25, 33). The GLP-1 analog liraglutide was previously shown to promote histological resolution of NASH and reduce metabolic dysfunction, insulin resistance, and lipotoxicity in patients living with the condition (1, 2). GLP-2 treatment has been shown to accelerate liver regeneration in mice following partial hepatectomy (16). GLP-1 and GLP-2 therapies have also been associated with improvements in intestinal epithelial barrier function, reducing local inflammation, which may be beneficial to resolving NASH (3, 7, 36). In the present study, the effects of the GLP-1 agonist, liraglutide, and the GLP-2 agonist teduglutide were investigated in mice fed an HFCD. Both liraglutide and teduglutide improved several characteristics of hepatic disease in this model. Therefore, this study is the first to describe the positive effects of teduglutide in a model of NAFLD/NASH, and the results indicate that teduglutide may have therapeutically beneficial effects for this indication in humans, possibly through improvements in intestinal barrier function, although further studies are required to confirm the mechanism of action. These results and previous studies on GLP analogs demonstrate the potential of TGR5 agonists like RDX8940 in the treatment of NASH.

RDX8940, shown here to act in the intestine with minimal systemic availability, did not inhibit gallbladder emptying in mice, nor did it have other previously observed toxicities of systemic TGR5 agonists. In recent years, studies of other intestine-targeted TGR5 agonists have been reported. These molecules show glucose-lowering activity in mouse models of diabetes, with reduced gallbladder-based side effects compared with absorbed TGR5 agonists (8, 14, 23). For example, the intestinally targeted TGR5a agonist 26a was investigated in *ob/ob* mice, which are hyperglycemic and hyperinsulinemic, by Cao et al. (8). When a single oral dose of 26a 100 mg/kg was administered to these mice, the compound was found at low concentrations in blood plasma and high concentrations in the intestine, like RDX8940, indicating minimal absorption. Unlike RDX8940 concentrations, 26a levels peaked in all intestinal tissues 2 h after dosing, when levels of 26a in plasma were in the 60–75 ng/ml range. However, direct comparison of RDX8940 and 26a in this regard is not possible, owing to the removal of chyme from intestinal tissue in the study by Cao et al.; therefore, levels of 26a in chyme were not taken into account (8). Plasma levels of aGLP-1 were sustained after 26a administration (100 mg/kg in female *ob/ob* mice), as was found with RDX8940 administration in the present study. In the study by Cao et al. (8), gallbladder area and bile weight were measured in overnight-fasted male *ob/ob* mice dosed with 26a 100 mg/kg once daily for 1 or 3 days to indicate the level of gallbladder filling. After a single 26a dose, gallbladder area and bile weight were similar to those recorded after dosing with vehicle, but after 3 days of 26a dosing, they had significantly increased. However, after dosing, these mice were given free access to food for 3 h, which causes gallbladder emptying, meaning that assessment of the gallbladder filling effect was compromised in this study. After administering 26a, 100 mg/kg orally once daily for 18 days in male *ob/ob* mice, levels of nonfasting and fasting glucose, triglycerides, and glycated hemoglobin significantly decreased compared with vehicle, consistent with the effects of RDX8940 observed in the present study.

In another study by Ma et al. (23), OL3, a minimally absorbed compound produced by linking linagliptin with the TGR5 agonist MN6, was dosed orally (150 mg/kg) in the Institute of Cancer Research (ICR) mice. Serum concentrations of OL3 decreased from 101.1 to 13.38 ng/ml 5.5 h after dosing, indicating minimal absorption. Compared with the control group, OL3 increased plasma aGLP-1 concentrations in treated mice by 26.4% (*P* < 0.05) 1.5 h after dosing, which followed 6 h of fasting. Comparisons between OL3 and RDX8940 in this regard are difficult to make owing to differences in dosing regimens. There were no significant changes in gallbladder volumes after OL3 dosing; however, as in the study by Cao et al. (8), gallbladder measurements were made after the OL3-dosed mice had been fed, preventing effective comparison with the present study.

Effects of another minimally absorbed TGR5 agonist, Compound 15c, were investigated by Duan et al. (14). Following oral dosing with Compound 15c (20 mg/kg) in rats, no detectable levels of the agent were found in plasma, although this pharmacokinetic study was conducted far below the effective dose of 150 mg/kg. At 4 h after dosing with Compound 15c (150 mg/kg) in ICR mice, there was a 97% increase in plasma aGLP-1 levels compared with vehicle. After dosing with Compound 15c (150 mg/kg) in overnight-fasted ICR mice, the gallbladder area increased by ~44%, which was not significantly different to the result obtained with controls. Again, gallbladder measurements were made after the mice had been refed, meaning that the initial filling effect after dosing was compromised, and the results cannot be compared directly with those of our study.

The effects of incretins on gallbladder physiology have highlighted the experimental challenge in deconvoluting the TGR5-dependent and -independent components of gallbladder filling. Yusta et al. (37) demonstrated that aGLP-2 has gallbladder filling effects that are GLP-2 receptor-dependent and not dependent on the presence of TGR5, and further that aGLP-2-mediated gallbladder filling was inhibited by coadministration of CCK8. A degree of background aGLP-2-mediated filling appears to be a factor in published studies of minimally absorbed TGR5 agonists where lipid meal (egg yolk) or CCK-mediated gallbladder emptying is not specifically measured. Differences in experimental methodology of previously published studies confound direct comparison of the different approaches to avoiding effects on the gallbladder. Future studies directed toward evaluation of the safety of minimally absorbed TGR5 agonists should employ experimental methodology that minimizes aGLP-2-mediated filling effects.

Overall, the data reported here show that RDX8940 is a potent, selective, and minimally systemic oral TGR5 agonist that induces GLP-1, GLP-2, and PYY secretion from mouse intestinal L cells, does not inhibit gallbladder emptying in mice, and improves liver steatosis and insulin sensitivity in a mouse model of NAFLD and mild insulin resistance. These findings warrant further studies to assess the potential therapeutic value of RDX8940 in patients with NAFLD/NASH.

## GRANTS

Medical writing support was provided by Dr. Tim Ellison of Oxford PharmaGenesis, funded by Ardelyx.

## DISCLOSURES

This study was funded by Ardelyx. All authors are current or former employees of Ardelyx.

## AUTHOR CONTRIBUTIONS

PDF, AJK, JSC, JGL, and JWJ conceived and designed the research. PDF, DR, JK, ZJ, SW, EB, TC, NB, DD, RJ, ML, MS, CWC, SK, KS, CL, SV, IH, KK performed experiments. PDF, AJK, KK, KO, CWC analyzed data. PDF, AJK, KK, KO, JGL interpreted results of experiments. PDF drafted the manuscript and prepared figures. AJK, JSC, JGL, KK and PDF edited and revised the manuscript. All authors approved the final version of the manuscript.
